# Hydrodynamic Analysis-Based Modeling and Experimental Verification of a New Water-Jet Thruster for an Amphibious Spherical Robot

**DOI:** 10.3390/s19020259

**Published:** 2019-01-10

**Authors:** Xihuan Hou, Shuxiang Guo, Liwei Shi, Huiming Xing, Yu Liu, Huikang Liu, Yao Hu, Debin Xia, Zan Li

**Affiliations:** 1 Key Laboratory of Convergence Medical Engineering System and Healthcare Technology, The Ministry of Industry and Information Technology, Beijing Institute of Technology, Beijing 100081, China; houxihuan@bit.edu.cn (X.H.); xinghuiming@bit.edu.cn (H.X.); liuyu_0827@bit.edu.cn (Y.L.); liuhuikang@bit.edu.cn (H.L.); huyao@bit.edu.cn (Y.H.); xiadebin@bit.edu.cn (D.X.); 3120180901@bit.edu.cn (Z.L.); 2 Faculty of Engineering, Kagawa University, 2217-20 Hayashicho, Takamatsu, Kagawa 761-0396, Japan

**Keywords:** amphibious spherical robot, water-jet thruster, thrust, hydrodynamic analysis, thrust model, Computation Fluid Dynamic (CFD)

## Abstract

Thrusters are the bottom actuators of the amphibious spherical robot, and play an important role in the motion control of these robots. To realize accurate motion control, a thrust model for a new water-jet thruster based on hydrodynamic analyses is proposed in this paper. First, the hydrodynamic characteristics of the new thruster were numerically analyzed using computational fluid dynamics (CFD) commercial software CFX. The moving reference frame (MRF) technique was utilized to simulate propeller rotation. In particular, the hydrodynamics of the thruster were studied not only in the axial flow but also in oblique flow. Then, the basic framework of the thrust model was built according to hydromechanics theory. Parameters in the basic framework were identified through the results of the hydrodynamic simulation. Finally, a series of relevant experiments were conducted to verify the accuracy of the thrust model. These proved that the thrust model-based simulation results agreed well with the experimental results. The maximum error between the experimental results and simulation results was only 7%, which indicates that the thrust model is precise enough to be utilized in the motion control of amphibious spherical robots.

## 1. Introduction

Amphibious robots have attracted increasing attention from researchers for various applications in complex environments [1]. They can be applied to pollution detection, terrain mapping, and for scouting potential approach lanes for amphibious naval operations in constricted areas [2,3,4]. The propulsion systems of amphibious robots play a vital role in achieving these wide applications in complex environments [5]. With the development of amphibious robots, many kinds of propulsive devices have been proposed and developed, for instance, wheel-propeller-fins [6], wheel-propeller-legs [7,8], and curved flipper legs [9]. However, devices such as the wheel-propeller-fin and wheel-propeller-leg are prone to breaking down because of abrasion and entanglement in weeds. Restricted thrust is an obvious defect of curved flipper legs. A propulsion mechanism with vectored water-jet thrusters was developed for the amphibious spherical robots by our team [10,11,12,13]. This kind of water-jet thruster ingests water from the inlet and then ejects water from the nozzle to generate a reaction (thrust) force for the robot. The propeller of this kind of water-jet thruster is installed inside a duct. The ducted structure is able to reduce the influence of the surrounding environment on the thrust. With the increasing number of multifunctional sensors, the size and weight of the robot have also increased. Previous water-jet thrusters were unable to provide enough thrust to drive the amphibious spherical robot. In order to overcome this defect, a new kind of water-jet thruster has been applied to the amphibious spherical robot IV (ASR-IV) [14,15].

The new water-jet thruster as the actuator of amphibious spherical robots is the basis of the control architecture of the whole system. Thrust prediction and modeling of the new thruster are required [16]. Approaches to determining the hydrodynamic force (thrust of a propeller, drag force of an underwater robot etc.) from currently published literature can be classified into theoretical methods, experimental methods and numerical predictive methods. In the first method, hydrodynamic force is analyzed based on hydromechanics theory such as the momentum conservation law and the Bernoulli Equation [17]. Blade element theory [18] is another relatively simple method to predict the thrust of a propeller [19]. The propeller is divided into a number of independent sections along its length. The thrust is summed over all sections. The disadvantage of theoretical methods is that concrete values for the model parameters cannot be determined, although these methods are able to provide a basic thrust model of a thruster. In experimental methods, the hydrodynamic force of thrusters is determined by some sensors in a cavitation tunnel or in a towing tank [20,21]. The prediction of the thrust of a propeller has been studied in [22], where experimental results were provided in static and dynamic conditions. The experimental devices consisted of a load cell, an electronic-optic, and an analog electromagnetic current meter. Hydrodynamic experiments for thrust prediction of a propeller-rudder system were conducted in the cylinder section of a cavitation tunnel [23]. A bollard pull test of a ducted thruster was carried out to predict the thrust when blades rotated in the forward direction and in the inverse direction [24]. A laser tachometer, a load cell, and a circular water channel tank facility were utilized in the test. The above-mentioned experimental methods can improve the accuracy of hydrodynamic force predictions, which requires expensive, high-precision and professional sensors. In numerical predictive methods, hydrodynamic forces are determined with computational fluid dynamics (CFD). In recent years, CFD simulation techniques have matured to achieve a high level of accuracy [25] and have been employed more and more extensively for analyzing the hydrodynamic force of underwater robots or propellers. One example can be found in [24], where the drag force of an underwater disk robot was predicted utilizing CFD and validation of the CFD results was carried out. The maximum error between the CFD results and experimental results was only 7%. The force of a propeller boss cap fin has been analyzed based on hydrodynamic simulations and the maximum deviation was only 2.5% [23]. Different turbulence models have been assessed to investigate the open water hydrodynamic characteristic of a marine propeller [26]. In this research, the numerical simulation was shown to be useful and suitable for acquiring the hydrodynamic characteristics of marine propellers. An estimation of the induced hydrodynamic periodic force of a marine propeller has been conducted via Fluent software [27]. This study reported that CFD is a reliable tool for estimating hydrodynamic force. In addition, other researchers [28,29] also applied CFD simulation techniques to the analysis of thrust of propellers and validated the CFD results. A weakness of this method is that it is not easy to determine the dynamic coupled relationships among the different factors that influence the hydrodynamic force.

Motivated by the aforementioned discussion, this paper aims at combining hydrodynamic simulations and the theory of hydromechanics to establish an accurate thrust model of a new thruster. As is known, a thruster is generally driven by an electrical DC motor. Therefore, thrust is simultaneously affected by the motor model, propeller design, and hydrodynamic factors [30]. In this paper, only the effects of the rotational speed of the propeller on thrust are analyzed, while the effects of the DC motor model on the rotational speed of the propeller are disregarded. Among the thruster design parameters, the effect of the nozzle diameter is discussed. As for hydrodynamic factors, the influence of the relative velocity of the robot and ambient flow on thrust was studied. The hydrodynamic characteristics of the new water-jet thruster were evaluated with respect to these three aspects. Furthermore, a theoretical model based on hydromechanics theory was developed to reveal the numerical relationship among the different factors influencing the thrust. Thereafter, the hydrodynamic simulation results were utilized to identify the unknown parameters of the theoretical model. Finally, to verify the accuracy of the thrust model, a series of experiments were systematically conducted.

The rest of this paper is organized as follows. Section 2 depicts the prototype of the amphibious spherical robot and the new water-jet thruster. Details of the hydrodynamic simulation are presented in Section 3. In Section 4, the hydrodynamic results of the new water-jet thruster are discussed in regard to three aspects. Based on the theory of hydromechanics, a theoretical thrust model of the water-jet thruster is established and the parameters of the thrust model are determined by the CFD simulation results in Section 5. The validation of the hydrodynamic simulations and the verification of the thrust model accuracy are discussed in Section 6. Finally, a conclusion is given in Section 7.

## 2. Related Works

### 2.1. Prototype of the Amphibious Spherical Robot IV

**Figure 1:**
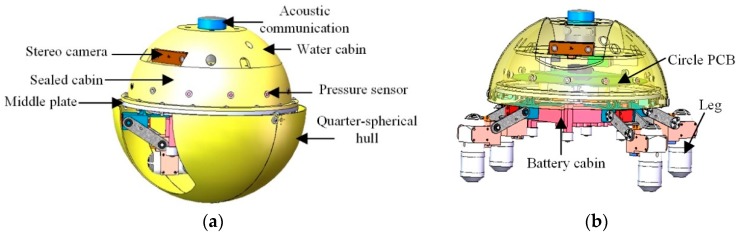
Structure of the amphibious spherical robot. (**a**) Overview of structure-spherical mode; (**b**) Overview of structure-hemispherical mode.

As introduced in reference [31], amphibious spherical robots have been proposed for missions or tasks in narrow spaces where traditional autonomous underwater vehicles (AUV) cannot operate. In order to improve its adaptability, a prototype of a new amphibious spherical robot, ASR-IV, has been proposed recently. The robot consists of a hemispheric upper hull, a circular middle plate, a legged water-jet composite driving mechanism, two quarter-spherical shells, a detachable battery cabin with 13,200 mAh and sensors, such as pressure sensors, an inertial measurement unit (IMU), and stereo cameras [14]. An overview of the structure of the robot is shown in Figure 1a. The hemispheric upper hull contains a top hull and a waterproof hull. The space between the top hull and the waterproof hull is the water storage cabin, which can adjust the buoyancy of the robot. The sealed cabin is made up of the waterproof hull and the middle plate, between which there is an O-ring to achieve seal. The circuit part is placed in the sealed cabin. The diameters of the upper and lower hemisphere of the amphibious spherical robot are 300 mm and 320 mm, respectively. The weight of the amphibious spherical robot is about 6.5 kg. The amphibious spherical robot can operate on land and in the water. In the water, the robot moves with two quarter-spherical shells closed, as visualized in Figure 1a. On land, the robot can walk with the two quarter-spherical shells opened as shown in Figure 1b.

### 2.2. Propulsion Mechanism of the Amphibious Spherical Robot IV

**Figure 2:**
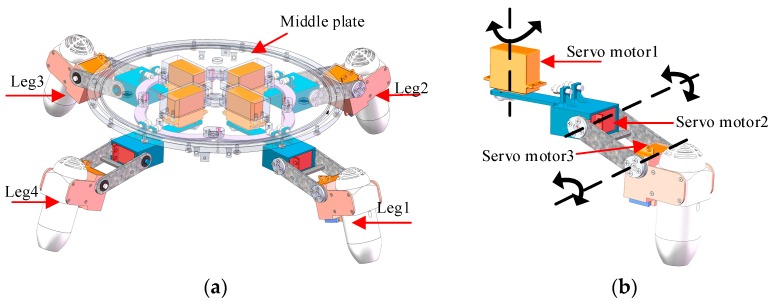
The mechanism of the propulsion system and the structure of the actuating unit. (**a**) Mechanism of propulsion system; (**b**) Structure of the actuating unit.

**Figure 3:**
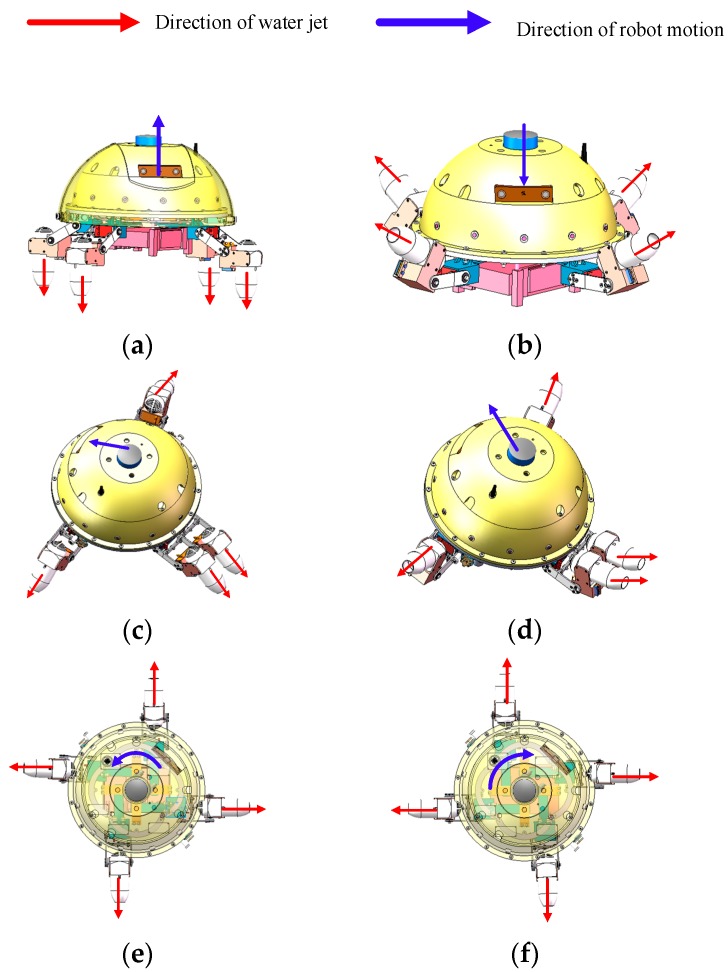
The mechanisms for different underwater motions. (**a**) Ascending motion; (**b**) Sinking motion; (**c**) Forward-up motion; (**d**) Forward-down motion; (**e**) Counter-clockwise rotation; (**f**) Clockwise rotation.

The propulsion mechanism is a crucial factor in achieving on-land and underwater locomotion. In order to observe the propulsion mechanism of the robot, only the propulsive part of the robot is shown in Figure 2. The propulsion set consists of four identical actuating units distributed around the circumference and installed under the middle plate. Each actuating unit consists of a new water-jet thruster and three servomotors giving each actuating unit three active degrees of freedom (DOFs). By changing the output angle of every servomotor and propulsive force of the new water-jet thrusters, the robot is able to achieve the motions of moving forward-up, forward-down, rotating, ascending and diving in the underwater environment, as shown in Figure 3. The red arrow represents the direction of the ejecting water. The blue arrow represents the moving direction of the robot. It is observed that changing the thrust values and directions of the water-jet thrusters can help the robot achieve different motions. Therefore, it is imperative to study the thrust model of the new water-jet thruster for the accurate motion control of the ASR-IV.

### 2.3. Prototype of the New Water-Jet Thruster

**Figure 4:**
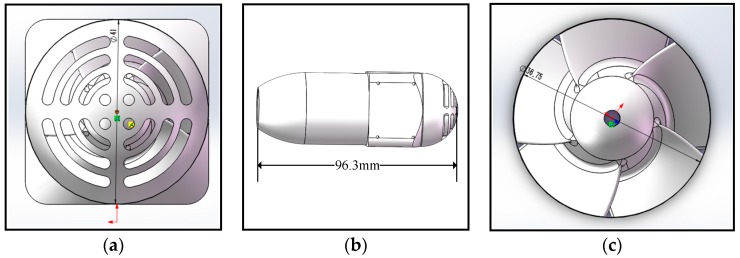
Size of some important parts of the new water-jet thruster. (**a**) Diameter of the dam-board; (**b**) Length of the thruster; (**c**) Diameter of the propeller.

In order to generate high thrust, a new water-jet thruster is applied to the amphibious spherical robot IV. The new water-jet thruster with a conical nozzle is an electromechanical device equipped with a motor and a propeller wrapped by a cylinder duct. It was made of 6061-T6 aluminum, which can bear heavier load than PVC, the material used for the previous thruster. The resistance of aluminum alloy is smaller than PVC, which will improve the efficiency of the power. The streamline shape of the thruster also reduces the resistance. The new thruster mainly consists of five parts: a motor, a propeller, an inlet dam-board, a cylinder duct, and a conical nozzle. The propeller has five blades. The cylinder duct wraps the propeller and the motor, which protects them from abrasion and entanglement. The conical nozzle produces higher thrust than a cylindrical nozzle. The geometric parameters of the key parts of the new thruster are listed in Figure 4 and Table 1. The effect that the nozzle diameter has on the thrust was studied through hydrodynamic simulations (Section 4), and the optimal diameter was determined.

## 3. Hydrodynamic Simulation Details of the Thruster

Hydrodynamic simulation by CFD is a common way to study a thruster because it clearly shows the hydrodynamic performances of the thruster. In addition, it can reflect changes in thrust due to various factors such as the rotational speed, the flow velocity and so on, which is important for the control system of the amphibious spherical robot. In this part, some of the necessary preparatory work for the hydrodynamic simulation is depicted in detail. All work in this paper was processed with CFX [23,24] associated with ANSYS WORKBENCH [32].

### 3.1. Establishment of the Computational Domain

**Figure 5:**
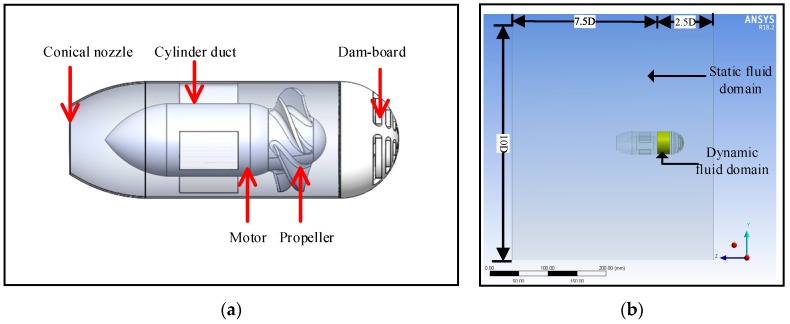
Simplified 3D model of the thruster and the computational domain. (**a**) Simplified 3D model of the water-jet thruster; (**b**) Computational domain.

A computational domain was established based on the simplified 3D model of the new water-jet thruster depicted in Figure 5a. The computational domain should be large enough to ensure that the wall cannot affect the hydrodynamic results [10]. Because the outline of the thruster is similar to a cylinder, the computational domain adopts a 3D cylindrical block. According to the guidelines in ITTC 7.5-03-02-03 [33] and ITTC 7.5-03-3-01 [34], the cylinder diameter is 10D, the length from the edge of the computational domain to the propeller center in the upstream is 2.5D, and the downstream side extends until 10D. D is the diameter of the propeller, as shown in Figure 4c. For the convenience of computation, the computational domain is divided into two zones. The one wrapping the propeller is set to be a cylinder body. The diameter is 1.1D and the length in the *Z*-axis of the region is equal to that of the propeller hub. This zone, known as the dynamic fluid domain (yellow-green area in Figure 5b) is utilized to simulate propeller rotation. The rest of the computational domain is treated as the static fluid domain, which is used to simulate the state of the open water. Figure 5b describes the computational domain.

### 3.2. Numerical Grids

**Figure 6:**
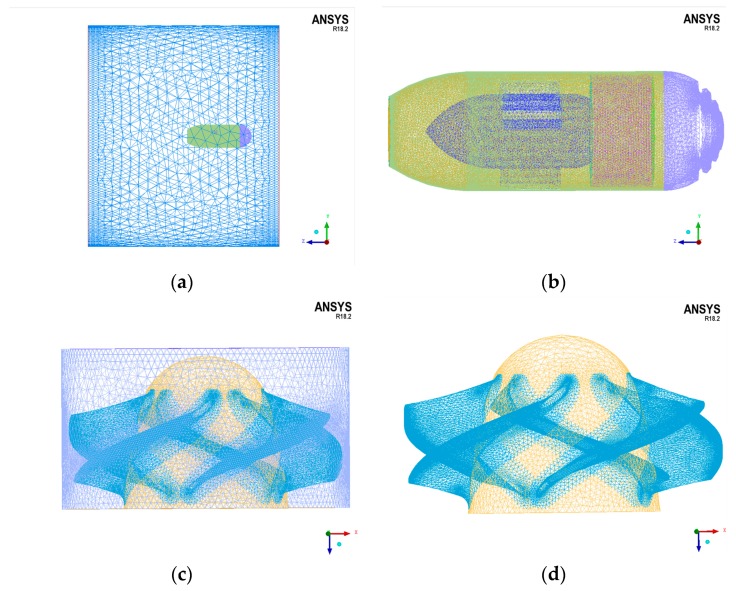
Surface grids of the static fluid domain and the dynamic fluid domain. (**a**) Surface grids of the static fluid domain; (**b**) Surface grids of the thruster in the static fluid domain; (**c**) Surface grids of the dynamic fluid domain; (**d**) Surface grids of the propeller in the dynamic fluid domain.

The hydrodynamic analysis is affected by the domain mesh density. In order to balance the accuracy and efficiency of the simulation, grid size varies according to domains. The mesh size of the static fluid domain is slightly larger, and the grid size of the dynamic fluid domain is relatively small, especially that of all blades in the water-jet thruster. Finally, all the grids are smoothed and some particular grids are adjusted in ICEM. The quality of all grids is improved up to 0.2 (grid quality indicator), which satisfies the technological requirements. Figure 6 shows the surface grids of the static fluid domain and the dynamic fluid domain. The total number of elements is 2,186,476, and that of the nodes is 684,221.

### 3.3. Solver Settings

Before starting the calculation, it is necessary to set boundary conditions for all domains. The inlet of the computational fluid domain is set as the velocity inlet. Because the flow out of the nozzle has no fixed direction in the real environment, the walls (except the inlet) are set as the opening boundary. Due to the lower computational cost, the moving reference frame (MRF) technique is widely used for numerical simulations of a rotating fluid domain in the CFD simulation. The rotational speed of the propeller is imposed by an MRF applied to the dynamic fluid domain [35] in the following simulations. Knowing the Reynolds number criterion, it is then possible to establish a shear stress transport (SST) turbulence model which has higher accuracy for flow-field simulation under turbulent conditions. The SST model is based on the k−w model and has the ability of automatic wall treatment. The wall treatment approach switches between Reynolds number formulation (i.e., direct resolution of the boundary layer) at low y+ values and a wall function approach at higher y+ values. The calculation can be terminated when the average residuals of RMS (root mean square) drop to $1\times 10^{-5}$ Moreover, the flow velocity of a point at the nozzle is set as a monitor to guarantee the convergence.

## 4. Simulation Analysis

This part analyzes the hydrodynamic characteristics of the new water-jet thruster in relation to three aspects. The first is the nozzle diameter. The second is the rotational speed of the propeller and the inlet velocity of the thruster. The third is the oblique angle of the ambient flow. Analysis of hydrodynamic characteristics mainly focuses on thrust. In addition, the pressure distribution on the blades and the velocity distribution in the thruster are also discussed for a better understanding of the hydrodynamic characteristics of the thruster.

Consistent with the majority of previous work on hydrodynamic analysis, a non-dimensional parameter J named as the advance coefficient is introduced to analyze the thrust of the propellers. This parameter can be expressed with the following equation

(1) J=vnD,

where v is the inlet velocity of water; n is the rotational speed of the propeller; D is the diameter of the propeller. Different values of the advance coefficient are obtained with a constant propeller rotational speed (3000 rpm) and a varying flow velocity.

### 4.1. Effect of Nozzle Diameter on Hydrodynamic Simulation Results

**Figure 7:**
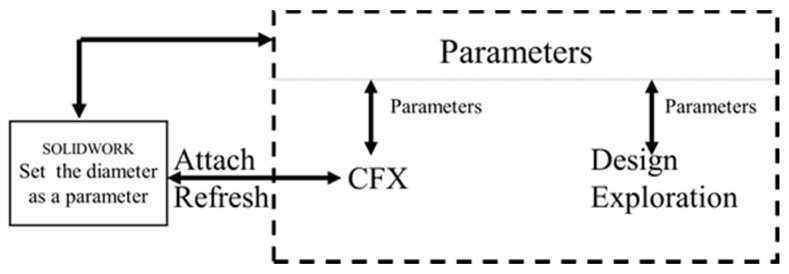
Workflow of optimization method.

In order to analyze the influence of the nozzle and find the most appropriate nozzle diameter, the thrust of the thruster with different nozzle diameters is predicted. It is inconvenient to build the 3D model of the water-jet thruster for each nozzle diameter in the simulation, therefore, SOLIDWORKS and ANSYS-Design Exploration [32] are combined to compute thrust in different cases. The nozzle diameter is set as a recognizable parameter in SOLIDWORKS. When the value of the parameter in ANSYS changes, the 3D model of the thruster in SOLIDWORKS updates automatically. Design Exploration is an optimization program that can obtain the optimal value of a target output parameter over a range of input parameters. In this project, the input parameter is the nozzle diameter, and the target output parameter is the thrust of the new thruster. The procedure of this method is described in Figure 7.

**Figure 8:**
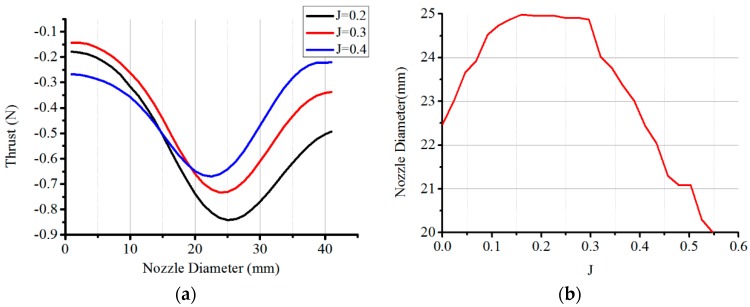
Thrust with nozzle diameter at three advance coefficients and optimal diameter of the nozzle. (**a**) Thrust with nozzle diameter; (**b**) Optimal diameter with advance coefficients.

The thrust generated by a conical nozzle is higher than that by a circular nozzle. As the duct diameter of the thruster shown in Figure 4a is 41 mm, the total permissible nozzle diameter is in the range of 1 mm to 41 mm. Figure 8a presents the thrust in the permissible nozzle diameter range for three advance coefficient settings. It can be seen that the thrust varies parabolically with the nozzle diameter for a given advance coefficient. In order to find the appropriate nozzle diameter with which the thruster can achieve maximum thrust, the design of experiments (DOE) method in the ANSYS-Design Exploration is utilized. Optimal values of the nozzle diameter with different advance coefficient settings are reported in Figure 8b. The optimal value varies from 20 mm to 25 mm, which is 12.2% of the total permissible nozzle diameter. The average of the optimal values is set as the diameter of the nozzle of the water-jet thruster.

### 4.2. Effect of Advance Coefficient on Hydrodynamic Simulation Results

#### 4.2.1. Effect of Advance Coefficient on Thrust

**Figure 9:**
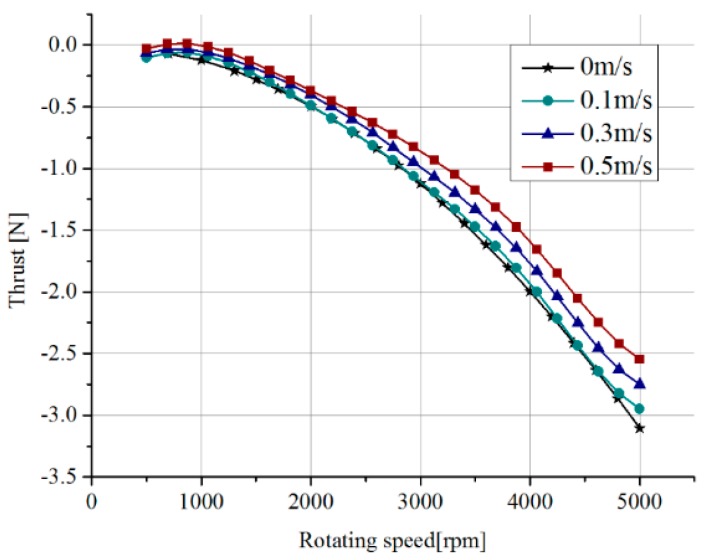
Thrust force at different rotating speeds and different inlet velocities.

**Figure 10:**
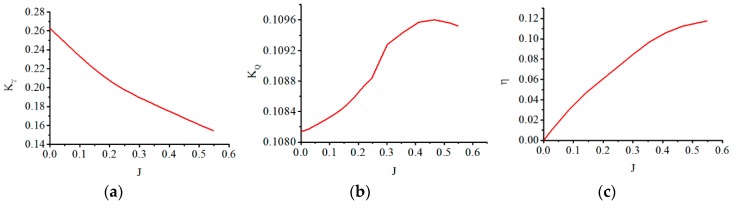
Thrust, torque and efficiency trends with the advance coefficient. (**a**) Thrust with advance coefficient J; (**b**) Torque with advance coefficient J; (**c**) Efficiency with advance coefficient J.

The results from the simulations conducted to analyze thrust at different rotational speeds and different inlet velocities are shown in Figure 9. Thrust increases as angular velocity increases at a certain inlet velocity. However, the effect of increasing inlet velocity is to reduce thrust. In addition, the effect of the angular velocity on thrust is much greater than that of the inlet velocity. Further, the thrust is analyzed with the advance coefficient. The largest velocity of ASR-IV (1 m/s) and the propeller rotational speed (3000 rpm) make the advance coefficient vary in the range of 0 to 0.54. Therefore, the hydrodynamic characteristic of the thruster is discussed only in this range. In order to keep dimensional agreement, the standard definition-based thrust coefficient [36] is adopted to analyze the effects of the advance coefficient. To be consistent with other hydrodynamic characteristic analysis [36], the torque coefficient and efficiency with the advance coefficient are also presented. The torque is generated by the drag that prevents blades rotating. Figure 10 illustrates the characteristic curves of the thruster in the advance coefficient range of 0 to 0.54. It is observed that the thrust coefficient decreases with the increase in the advance coefficient. This trend agrees with that reflected in Figure 9. The torque coefficient has little growth with the advance coefficient. The efficiency of the thruster gradually increases in the advance coefficient range. The trends of the thrust coefficient and efficiency are almost consistent with that of the propeller VP1304 in [36] while, the trend of the torque coefficient is the opposite, which is caused by the smaller drag force on blades.

#### 4.2.2. Pressure Distribution on Blades

**Figure 11:**
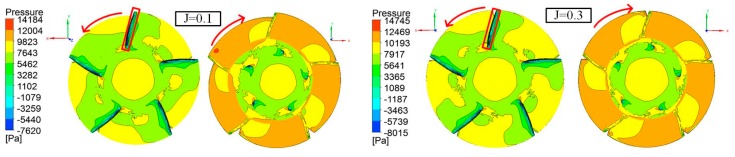
Pressure contours on the pressure side (**right**) and the suction side (**left**) at J=0.1 and J=0.3.

Figure 11 depicts contour plots of the pressure distribution of the propeller for two advance coefficient settings of 0.1 and 0.3. The red arrow indicates the rotating direction of the thruster and the red rectangle indicates the leading edge of a blade. It is found that pressure on all blades becomes greater with increases in the advance coefficient according to the left legend. On the pressure side, the influences of the advance coefficient on pressure are minimal, however, the influence is significant on the suction side. When the advance coefficient value is larger, the yellow area (higher pressure) on the pressure side becomes larger.

#### 4.2.3. Effects of Advance Coefficient on Flow in the Thruster

**Figure 12:**
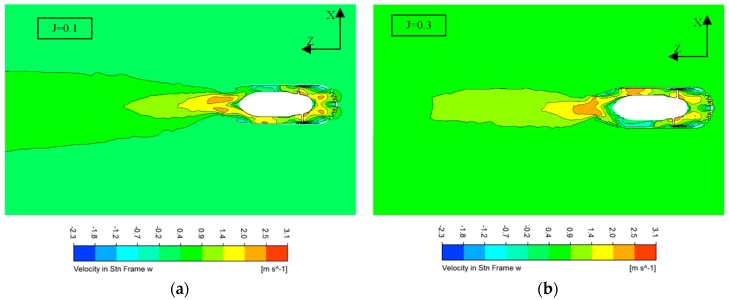
Stream velocity Z on the X-Z plane. (**a**) Stream velocity z at J=0.1; (**b**) Stream velocity z at J=0.3.

In the X-Z section, velocity-Z for two different advance coefficients is shown in Figure 12. It is obvious that the length of the wake flow stretching is short for a larger advance coefficient. Longer distance reflects higher thrust.

### 4.3. Effect of the Angle of Oblique Flow on Hydrodynamic Simulation Results

#### 4.3.1. Effect of the Angle on Thrust

**Figure 13:**
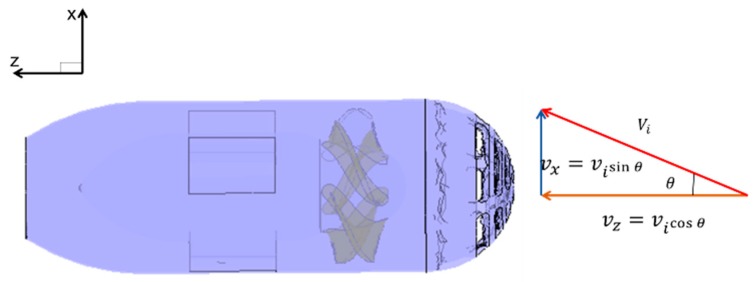
Diagram of oblique flow.

There are two methods to achieve hydrodynamic simulations of the water-jet thruster in oblique flow. The first is to adjust the angle of the thruster model. The other is to change the incoming flow velocity component [37]. The simulations in this section applied the latter. The advantage is that only one calculation model needs to be established whether the working condition is axis flow or random oblique flow. A sketch of the water-jet thruster in the inclined flow is shown in Figure 13.

**Figure 14:**
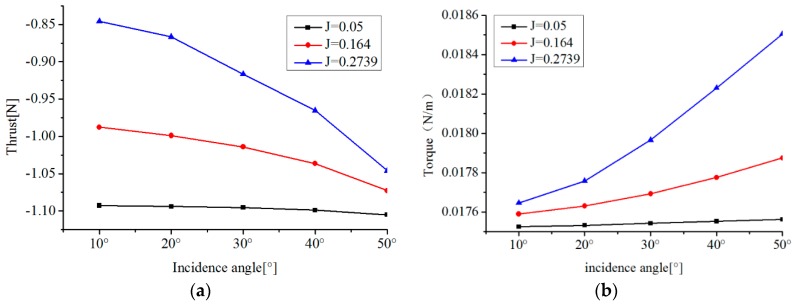
Characteristics of thrust and torque at different incidence angles. (**a**) Thrust at different incidence angles; (**b**) Torque at different incidence angles.

In order to investigate the performance of the thruster in oblique flow, thrust is calculated in the oblique angle range of 10°–50° for three advance coefficient values (0.05, 0164 and 0.2739). Moreover, torque is also calculated for a comprehensive hydrodynamic analysis. The characteristics of the thrust and torque at different oblique angles are drawn in Figure 14. For a given advance coefficient, thrust and torque increase with the increase of the incidence angle. The increasing rate of the thrust and the torque with the incidence angle is higher for the larger advance coefficient. At a fixed incidence angle, thrust is larger for a lower advance coefficient, which agrees with that in axis flow, while changes of torque are very slight, measured by 0.0001.

**Figure 15:**
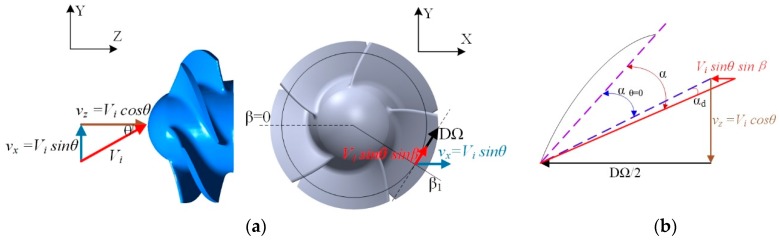
Sketch of blade element-based analysis. (**a**) Definition of blade section angle; (**b**) Velocity polygon on the blade section.

Furthermore, the oblique inflow velocity $V_{i}$ is divided into two components on the thruster’s coordinate system, axial velocity $v_{z}$ and tangential velocity $v_{x}$ as depicted in Figure 13. In order to dissect the effects of $v_{z}$ and $v_{x}$ on thrust, further analysis based on Blade Elementary [19] is shown on a blade. The coming flow of a blade section consists of the axial velocity component $v_{z}$ and the circumferential velocity component $v_{t}$ as shown in Figure 15. $v_{z}$ and $v_{t}$ are defined as the followings:

(2) $$v_{z}=V_{i}\cos\theta ,$$

(3) $$v_{t}=\frac{D\Omega }{2}-v_{x}\sin\theta =D\Omega -V_{i}\sin\theta \sin\beta ,$$

where, *D* is the diameter of the propeller, Ω is the propeller rotation angular velocity, θ is the oblique flow angle, and β is the circumferential position of a blade section. The angle of attack of a blade section in oblique flow is defined as the following:

(4) $$\alpha =\Theta -\arctan(\frac{v_{z}}{v_{t}}),$$

where, Θ is the pitch angle of the blade section. $\alpha_{d}$ in Figure 15b is defined as follows,

(5) $$\alpha_{d}=\alpha -\alpha_{\beta =0},$$

where, $\alpha_{\beta =0}$ is the attack angle of the blade section in axial flow. $\alpha_{d}$ is the difference of attack angle, which is caused by the tangential velocity component $v_{x}$. According to Equation (5), the thrust of the thruster in oblique flow is caused by two angles, $\alpha_{\beta =0}$ and $\alpha_{d}$. In other words, the thrust of the thruster in oblique flow is induced by the axial velocity component $v_{z}$ and the tangential velocity component $v_{x}$. Thrust in oblique flow can be defined as follows:

(6) $$T=T_{1}+T_{2},$$

where, T is the total thrust of the thruster; $T_{1}$ is the thrust component that is generated by the axial velocity component $v_{z}$; $T_{2}$ is the other thrust component generated by the tangential velocity component $v_{x}$. $T_{1}$ can be calculated through $K_{T}$ as shown in Figure 10a. $T_{2}$ is calculated according to Equation (6). For the convenience of computing $T_{2}$, the advance coefficient in this simulation is defined as

(7) $$J=\frac{v_{z}}{D\Omega }=\frac{v_{i}\cos\theta }{D\Omega },$$

**Figure 16:**
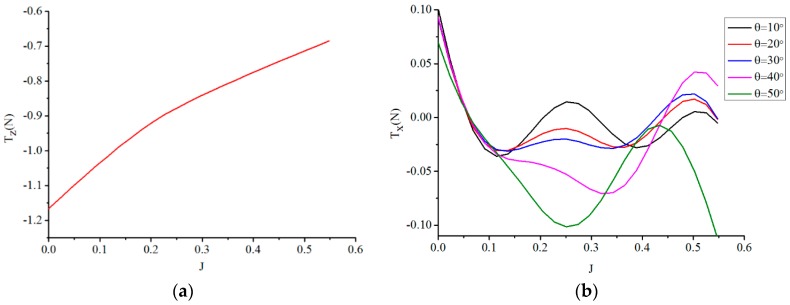
Thrust generated by the axial component and tangential component of velocity. (**a**) Thrust generated by the axial component of velocity; (**b**) Thrust generated by the tangential component of velocity.

$T_{1}$, $T_{2}$ are plotted with the advance coefficient at different oblique angles shown in Figure 16a,b, respectively. It is obvious that $T_{2}$ is very small compared to $T_{1}$ and the maximum of $T_{2}$ is close to −0.1 for five given oblique angles. This result demonstrates that the axial velocity component has the most important effect on the thrust of the thruster. The tangential component of oblique flow velocity has little influence on thrust. Therefore, it is allowable to neglect $T_{2}$. In oblique flow, thrust of the new thruster can be regarded as the force generated by the axial component of inlet velocity only.

#### 4.3.2. Effect of the Oblique Flow Angle on Pressure Distribution

**Figure 17:**
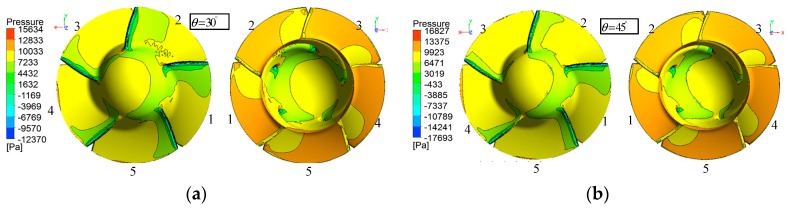
Pressure contours on the propeller under different oblique angles at J=0.4. (**a**) Pressure contours on pressure side (**right**) and suction side (**left**) at θ=30°; (**b**,**d**) Pressure contours on pressure side (**right**) and suction side (**left**) at θ=45°.

Figure 17 shows the pressure distribution on the suction side and the pressure side of blades at two different incidence angles for the case of J=0.4. Comparing the suction side pressure distribution in Figure 17a to that in Figure 17b, there exist clear differences on all blades except blade1. The difference is that low-pressure transition areas (green zone) decrease as the oblique angle increases. Observing the pressure side in Figure 17a,b, the pressure distribution shows little disproportion and the low-pressure transition areas (yellow areas) on every blade decrease with oblique angle increase. In a whole, the increase of oblique angle makes the pressure distribution more unsteady.

#### 4.3.3. Effect of the Angle on Flow Inside the Thruster

**Figure 18:**
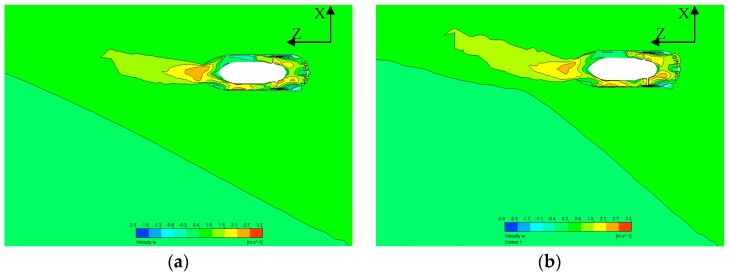
Stream velocity z on the X-Z plane at J=0.4. (**a**) Stream velocity z at J=0.4, θ=30°; (**b**) Stream velocity z at J=0.4, θ=45°.

**Figure 19:**
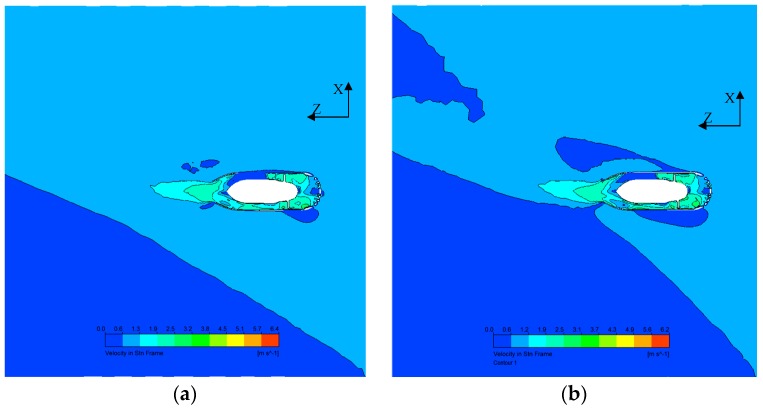
Resultant velocity on the X-Z plane at J=0.4. (**a**) Resultant velocity at J=0.4, θ=30°; (**b**) Resultant velocity at J=0.4, θ=45°.

In order to investigate water flow in the thruster and wake flow in oblique flow, velocity-Z in the X-Z section for two cases are plotted in Figure 18. The wake flow of the thruster is oblique when it works in inclined water flow. The oblique angle at the nozzle is smaller than that of the initial water flow, which proves that a long duct can reduce the effect of the oblique flow. Velocity contours in a resultant frame in the X-Z section are shown in Figure 19. It is easily seen that the effects of the thruster on the surrounding water area are much greater with the increase in the oblique angle.

## 5. Model of the New Water-Jet Thruster

Because thrusters are the bottom actuators of the amphibious spherical robot, an accurate thrust model of the thruster is required to realize accurate motion control. In this section, the basic model of the thruster is established based on the theory of hydromechanics and the model parameters are identified based on the aforementioned hydrodynamic simulation results.

### 5.1. Basic Model of the New Water-Jet Thruster

**Figure 20:**
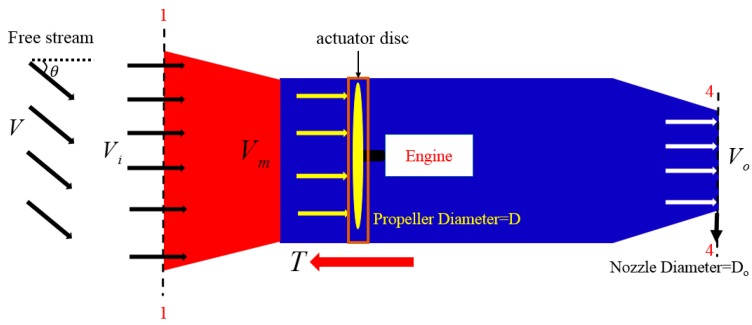
Thrust nomenclature system.

In the following analysis of thrust, gravity will be neglected. The control volume consists of the stream-duct of fluid that passes through the propeller plane area. Figure 20 depicts the flow model of the internal situation of the thruster. V is the ambient flow velocity. $V_{i}=V\cos\theta$ is the axial component of the ambient flow. θ is the ambient flow oblique angle relative to the rotating axis of the propeller. According to the analysis in Section 4.3, the thrust of the new water-jet thruster in oblique flow is simplified as the force generated by the axial component of inlet velocity. Therefore, $V_{i}$ is considered directly in the process of modeling thrust.

Momentum theory (disk actuator theory) [38,39] is a theory describing a mathematical model of an ideal actuator disc, such as a propeller. According to momentum theory, the propeller inside the water-jet thruster is thought of a thin actuator disc with an infinite number of blades. There is no rotation imparted to the flow by the actuator disc. The actuator disc imparts momentum to the incoming flow. With the pressure being equal at 1-1 and 4-4, as shown in Figure 20, it does not contribute to the surface force. There is no other force in the horizontal direction that acts upon the control volume except the thrust. Therefore, thrust is equal to the difference between the outflow momentum and the inflow momentum per unit time. Accordingly,

(8) $$T=\dot{m}\left(V_{o}-V_{i}\right),$$

where, T is the total thrust of the thruster; $V_{i}$ is the axial component of the free stream velocity V. $\dot{m}$ is the rate of mass. An application of the continuity equation gives,

(9) $$\frac{\pi }{4}D^{2}V_{m}=\frac{\pi }{4}D_{o}^{2}V_{o},$$

where, $V_{m}$ is the linear sum of $V_{i}$ and DΩ [17]. Thrust is derived as follows:

(10) $$T=\frac{\pi }{4}D^{2}\rho \alpha^{2}\left[\left(k_{1}^{2}-\frac{k_{1}}{\alpha^{2}}\right){\left(V\cos\theta \right)}^{2}+(2k_{1}k_{2}-\frac{k_{2}}{\alpha^{2}})(V\cos\theta )D\Omega +k_{2}^{2}D^{2}\Omega^{2}\right],$$

where, $k_{1}$, $k_{2}$ are constants, Ω is the rotational speed of the propeller, and $\alpha =\frac{D}{D_{o}}$. The non-dimensional thrust coefficient $K_{T}(J)$ is represented as the following,

(11) $$\begin{array}{l} K_{T}\left(J\right)=\left[k_{1}^{\prime }{\left(\frac{V\cos\theta }{D\Omega }\right)}^{2}+k_{2}^{\prime }\frac{V\cos\theta }{D\Omega }+k_{3}^{\prime }\right] \end{array}$$

where, $J=\frac{V\cos\theta }{D\Omega }$ is the advance coefficient, which considers the ambient flow oblique angle. Thrust can be expressed with the thrust coefficient $K_{T}(J)$ as described in Equation (12). Unlike the common model of a marine propeller, the above thrust model considers the ambient flow oblique angle and the nozzle diameter.

(12) $$T=\frac{\pi }{4}\rho \alpha^{2}D^{4}\Omega^{2}K_{T}(J),$$

### 5.2. Identifying the Parameters of the Thrust Model

According to Equation (12), another expression of $K_{T}(J)$ is shown in Equation (13),

(13) $$K_{T}(J)=\frac{T}{\rho D^{4}\Omega^{2}}\times (\frac{4}{\pi \alpha^{2}})$$

**Figure 21:**
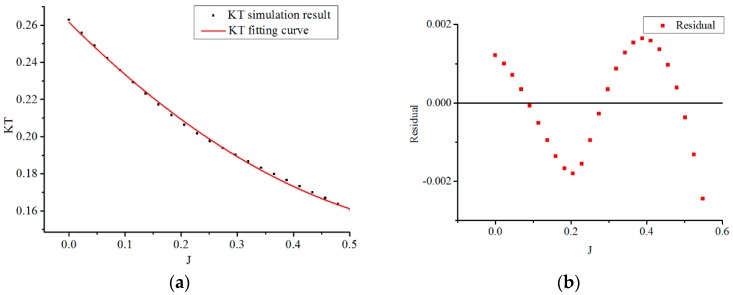
Fitting curve of KT with different advance coefficients. (**a**) Fitting curve of KT; (**b**) Residual of fitting curve.

According to the values of $K_{T}(J)$ with advance coefficient J shown in Figure 10a, a fitting curve of $K_{T}(J)$ in Figure 21 is approximated with the polynomial functions by MATLAB software as

(14) $$\frac{\pi \alpha^{2}}{4}K_{T}=0.20207J^{2}-0.30192J+0.350165,$$

Then, the thrust model of the new water-jet thruster can be depicted as

(15) $$\begin{array}{ll} T & =\frac{\pi }{4}\rho \alpha^{2}D^{4}\Omega^{2}K_{T}(J)\\ & =\rho D^{4}\Omega^{2}(0.20207J^{2}-0.30192J+0.350165) \end{array}$$

## 6. Verification of the Thrust Model of the New Water-Jet Thruster

The experiments are described in this section. In order to verify the accuracy of the thrust model, firstly, the numerical simulation results based on above settings were validated. Furthermore, the thrust model-based simulation results were compared to the experimental results.

### 6.1. Experimental Design and Preparation

**Figure 22:**
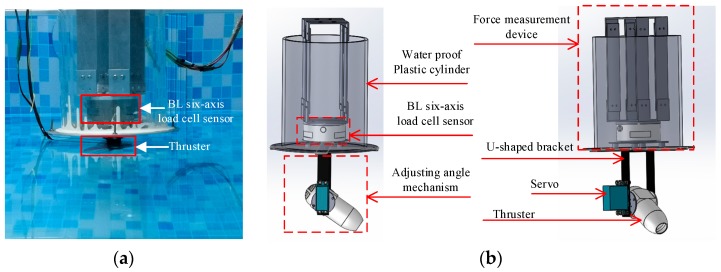
Experimental device. (**a**) Prototype of the experimental device; (**b**) Sketch of force measurement and adjusting angle mechanism.

The experimental system consists of two parts, a force measurement device and an adjusting angle mechanism. The practical experimental devices are visualized in Figure 22a. In order to clearly visualize the devices, Figure 22b depicts the 3D structure of the force measurement device and the angle adjustment mechanism. A BL six-axis load cell sensor is applied in the experiments. The sensor can detect forces and torques in the X, Y, and Z direction simultaneously [40]. Different standard weight commodities are utilized to calibrate the load cell sensor many times, which ensures the reliability of the experimental data. To protect the cell sensor from water, a plastic cylinder with 5 mm thickness was designed as shown in Figure 21b. Considering the influences of thruster oblique angle relative to the water flow, the angle adjustment mechanism was designed. The oblique angle of the thruster can be adjusted by a servo installed on the side of a U-shaped bracket. The outputting angle of the servo varies 10° every 1% duty cycle of the PWM, and the angle range of this servo is 160°. In addition, a water suction pump is utilized to provide a certain velocity of water flow. Because the water-jet thruster in this paper is small, the water flow passing through the water-jet thruster is assumed to be uniform. The distance between the water pump and the water-jet thruster was adjusted to achieve different flow velocities.

### 6.2. Validation of the Numerical Simulation

As the parameters of the thrust model were determined by the hydrodynamic simulation results in Section 4, validation of the hydrodynamic simulation results is primary to verify the accuracy of the thrust model of the new thruster. Generally, the mesh uncertainty is the main source of uncertainty in a hydrodynamic simulation. The numerical uncertainty is evaluated through a series of simulations corresponding to different mesh densities. According to ITTC 7.5-03-01-01 [41], five sets of grids are adopted and the mesh refinement ratio is $\sqrt{2}$. The thrust of the water-jet thruster based on hydrodynamic simulations is calculated as a comparable parameter with the experimental result. The results are listed in Table 2. It can be seen that the thrust gradually increases then does not change dramatically with different mesh densities, which indicates that the grids of MESH 3, MESH 4 and MESH 5 are adequate for producing reasonable results. The hydrodynamic simulation cases in Section 4 are based on MESH 5, which prove that the simulation data applied to the thrust model are reliable.

### 6.3. Experimental Verification of the Thrust Model

To verify the accuracy of the thrust model, two experiments were designed to measure the thrust of the new thruster. First, the thrust was tested for different rotational speeds at different velocities of axial water flow. Second, when the velocity of oblique water flow was maintained at 0.22 m/s, the thrust was tested for different rotational speeds at different oblique angles.

#### 6.3.1. Verification of Thrust Model at Different Velocities of Flow Water

**Figure 23:**
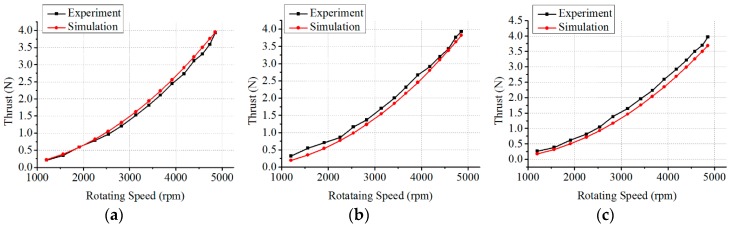
Thrust comparison of simulation results and experimental results. (**a**) Thrust comparison at 0.1 m/s; (**b**) Thrust comparison at 0.22 m/s; (**c**) Thrust comparison at 0.35 m/s.

**Figure 24:**
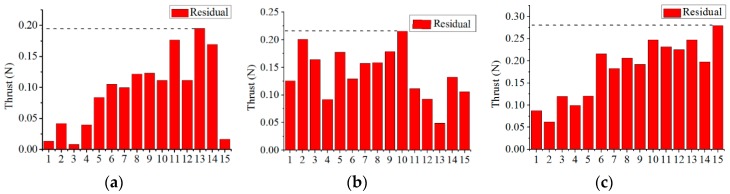
Residual between simulation results and experimental results. (**a**) Thrust residual at 0.1 m/s; (**b**) Thrust residual at 0.22 m/s; (**c**) Thrust residual at 0.35 m/s.

In this experiment, thrust is tested at three different flow velocities (0.1 m/s, 0.22 m/s, 0.35 m/s). For each velocity of water flow, the thrust is measured at fifteen different rotational speeds of the propeller. In each situation, data are collected 506 times, and the data is processed with a Kalman filter to obtain the final experimental result. In addition, the thruster must be kept horizontal in this experiment. The thrust based on the thrust model developed in Section 5 is calculated in the same conditions. The experimental results and thrust model-based simulation results for three different flow velocities are plotted in Figure 23a–c, respectively. According to Figure 23, the thrust model-based simulation results and experimental results are almost consistent. Meanwhile, residuals are depicted for three different flow velocities in Figure 24. Residuals refer to the absolute values of the difference between the experimental data and thrust model-based simulation results. In this Figure, the horizontal axis indicates fifteen sampling points of rotational speeds and the dashed lines indicate the maximum of the residuals. It can be observed that the maximum residual reaches 0.28 N, accounting for 7% of the experimental result at the rotational speed of 4853 rpm and flow velocity of 0.35 m/s. The average residuals of the thrust are 0.09 N, 0.14 N and 0.18 N for three flow velocities of 0.1 m/s, 0.22 m/s and 0.35 m/s, respectively.

#### 6.3.2. Verification of the Thrust Model under Different Oblique Angles

**Figure 25:**
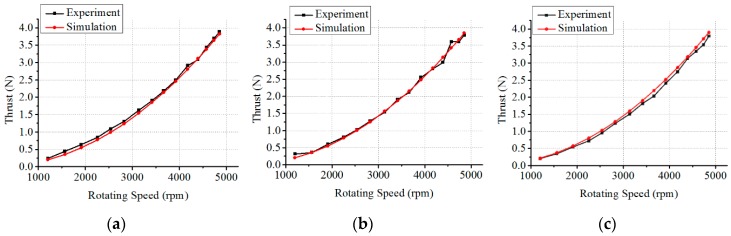
Thrust comparison of simulation results against experimental results under different oblique angles. (**a**) Thrust comparison at 10°; (**b**) Thrust comparison at 30°; (**c**) Thrust comparison at 50°.

**Figure 26:**
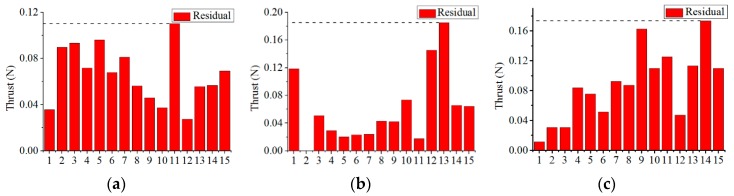
Residual between simulation results and experimental results. (**a**) Thrust residual at 10°; (**b**) Thrust residual at 30°; (**c**) Thrust residual at 50°.

In this experiment, the flow velocity is kept as 0.22 m/s. The oblique angle of the thruster relative to horizontal is adjusted by the adjusting angle mechanism. The thrust is investigated at oblique angles of 10°, 30°, 50°. For each oblique angle, the thrust is measured for fifteen different rotational speeds of the propeller. In order to get accurate results, data is collected 506 times in each condition, and a Kalman filter is utilized to filter the results. The experimental results and thrust model-based simulation results at three different oblique angles are shown in Figure 25a–c, respectively. It can be observed that most simulation results are consistent with the experimental results. Residuals between experimental results and simulation results based on the thrust model are plotted in Figure 26. The maximum residual is 0.19 N accounting for 5.3% of the experimental result at the rotational speed of 4574 rpm and under the oblique angle of 30°. The average residuals of the thrust at three oblique angles of 10°, 30°, 50° are 0.07 N, 0.06 N and 0.09 N, respectively.

### 6.4. Discussion

Due to the experimental environment and equipment, there were some errors in the experiments. A large amount of experimental data can reduce the error to a negligible range. The thrust model-based simulation results were compared to the experimental results and based on these comparisons, the maximum error of the thrust was 0.28 N, accounting for 7% of the experimental result. The thrust is the driving force of the amphibious spherical robot weighing 6.5 kg. The maximum thrust error, 0.28 N, has little effect on the motion of the robot. These errors are small enough to validate the thrust model developed in this paper. Moreover, the accuracy of the parameters of the thrust model identified based on hydrodynamic analysis is convincing. Furthermore, this method of combining hydromechanics theory and hydrodynamic simulations is available for modeling of the water-jet thruster. Compared to methods based only on experiments, this modeling method has an advantage because it is very time efficient, and it ensures the basic structure of the thrust model according to theory and then utilizes enough hydrodynamic simulation results to identify the parameters of the model.

## 7. Conclusions

The propulsion mechanism of the amphibious spherical robot plays a vital role in achieving different motions. The new water-jet thruster is the most important component of the propulsion mechanism. In order to achieve accurate motion control, this paper has focused on building the hydrodynamic simulation-based thrust model of the thruster. First, according to analyses of the simulation results, the thrust of the water-jet thruster decreases with an increase in the advance coefficient in axis flow. When the thruster operates in oblique flow, simulation results showed that the tangential velocity component of oblique water flow has little effects on thrust. The effects of the axial velocity component are the same as that of the axial-symmetric case only. Second, this paper developed the thrust model based on hydrodynamics theory and identified the parameters of the theoretical model based on hydrodynamic simulation data. Simulation results based on this thrust model agreed well with the experimental results. The maximum error between simulation results and experimental results is only 7.0%. This is small enough to verify the accuracy of the thrust model of the water-jet thruster.

The thrust model of the thruster suggested in this paper lays the foundation for building an accurate motion controller for the amphibious robot. Further research will focus on building a model of the amphibious spherical robot to achieve accurate motion control.

## Figures and Tables

**Table 1:** Thruster geometric parameters.

Parameter	Value
Propeller diameter	D = 36.5 mm
Number of blades	Z = 5
Thruster length	L = 96.3 mm
Rated power of motor	80 W

**Table 2:** Mesh sensitivity study.

	Total Cells (10^3^)	Rotational Region Cells (10^3^)	Thrust (N)	Difference %
Experiment			2.424	
MESH1	32.313	13.888	1.983	18.19
MESH2	58.138	24.316	2.118	12.62
MESH3	118.985	45.393	2.316	4.46
MESH4	173.321	96.384	2.358	2.72
MESH5	218.648	121.688	2.367	2.35
